# Anatomical and quantitative analysis of safe entry zones to the lower brainstem through the far-lateral approach

**DOI:** 10.1007/s10143-026-04171-7

**Published:** 2026-03-03

**Authors:** Daniel Dutra Cavalcanti, Eberval Gadelha Figueiredo, Helbert de Oliveira Manduca Palmiero, Mark C. Preul, Robert F. Spetzler

**Affiliations:** 1 Cerebrovascular and Endovascular Neurosurgery, Ayer Neuroscience Institute, St. Vincent’s Medical Center, Hartford HealthCare Medical Group, Bridgeport, CT USA; 2 Division of Neurosurgery, University of São Paulo Medical School, Dr. Enéas de Carvalho Aguiar Ave., 255, Room 5083, São Paulo, 05403-000 Brazil; 3 Barrow Neurological Institute, Phoenix, AZ USA

**Keywords:** Brainstem surgery, Medulla oblongata, Safe entry zones, Far-lateral approach, Microsurgical anatomy, Neuronavigation

## Abstract

Safe entry zones (SEZs) in the medulla oblongata offer consistent anatomical corridors for accessing intrinsic lesions while minimizing damage to nuclei and tracts. Data quantifying the geometric parameters of surgical exposure to the lower brainstem are limited. This study aims to anatomically and quantitatively analyze the exposure area, working angles, and linear extent of access to the medullary SEZs via the far-lateral approach. Ten formalin-fixed human cadaveric heads were dissected using simulated operative positioning. The far-lateral approaches were performed under neuronavigation, with coordinates of seven standardized landmarks on the brainstem surface recorded. Customized software calculated exposure areas, horizontal and vertical working angles, and the vertical linear extent of exposure. The far-lateral route optimized exposure of the ventrolateral medulla and olivary zone. The average exposed area for the far-lateral approach was 856.8 ± 139.7 mm². The horizontal and vertical working angles toward the olivary zone were 40.8 ± 10.2° and 54.8 ± 6.8°, respectively, with a vertical linear extent of exposure of 60.0 ± 6.6 mm. These data quantitatively characterize the operative field and instrument maneuverability provided by each approach. This study offers a quantitative anatomical framework for the far-lateral approach to the ventrolateral medulla, outlining objective parameters of exposure area, working angles, and vertical linear extent to the olivary safe entry zone. By converting microsurgical medullary anatomy into measurable operative geometry, these data support the far-lateral route as an effective and reproducible corridor for accessing intrinsic medullary lesions and could improve preoperative planning, trajectory selection, and surgical safety.

## Introduction

The brainstem compresses a dense collection of nuclei and long tracts into a small, thumb-sized structure, so even minimal manipulation carries a risk of significant neurological complications. The medulla oblongata—the caudal part of the brainstem located between the pons and the foramen magnum—houses reticular networks crucial for cardiorespiratory control, is crossed by compact ascending and descending pathways, and is associated with seven cranial nerves at the medullary and pontomedullary levels (VI–XII). Advances in neuroimaging, microsurgical techniques, skull-base approaches, and neurophysiological monitoring have gradually increased the operability of intrinsic brainstem lesions, particularly cavernous malformations and focal gliomas. Current series report acceptable outcomes when approaches and trajectories are carefully chosen [1–4].

When lesions do not present to a pial or ependymal surface, “safe entry zones” (SEZs) guide the points and directions for parenchymal transgression through areas where eloquent nuclei/tracts and perforators are relatively sparse. At the medullary level, several classically described surface corridors (e.g., the anterolateral/pre-olivary sulcus, regions along the lateral medullary surface adjacent to lower cranial nerve rootlets, and the posterior median sulcus) have served as anatomical references for parenchymal entry. Importantly, these are conditional corridors: the selection of an entry point must be individualized based on the lesion’s pathomorphology (growth pattern, pial/ependymal presentation, displacement of tracts/nuclei, and relationship to surface landmarks) and refined using image guidance and neurophysiological monitoring [1, 2]. The choice of exposure depends on the lesion’s topography: a midline suboccipital approach provides direct views of the posterior medullary sulci, while a far-lateral route—often involving partial removal of the C1 arch and limited occipital condyle drilling—optimizes access to the ventrolateral medulla and the olivary region [2, 5, 6]. Although early evidence for specific SEZs was based on cadaveric and small clinical series, larger modern cohorts have confirmed their safety when used to reach deep lesions with careful trajectory planning and monitoring [1, 3, 4, 6].

The present study anatomically defines and quantifies the exposure to the lower brainstem achieved through the far-lateral approach, focusing on the area of exposure and the horizontal and vertical working angles to the ventrolateral medulla (olivary region). By combining qualitative anatomical description with quantitative measurements, we aim to inform preoperative planning and the choice of approach/SEZ for intramedullary medullary pathology, complementing the existing clinical literature on SEZ safety and outcomes.

## Methods

This study was designed as an anatomical and quantitative cadaveric investigation of the far-lateral approach to the lower brainstem. The methodological framework and anatomical definitions were informed by established microsurgical literature on brainstem SEZs and skull base approaches. However, no formal systematic literature review or meta-analysis was conducted as part of the study design, but rather to find the anatomical foundations and microsurgical techniques of the far-lateral approach to the medulla oblongata. Following this conceptual grounding, the methodology focused on cadaveric dissection and neuronavigation-based quantitative measurements of exposure area, working angles, and vertical linear extent achieved through the far-lateral corridor.

### Anatomical dissection

Anatomical dissections were conducted on 10 human cadaveric heads fixed in formalin and injected with silicone to carefully illustrate the microsurgical anatomy of the lower brainstem and the technical details of the far-lateral approach. All dissections were performed at the *Barrow Neurological Institute Skull Base Laboratory* (St. Joseph’s Hospital and Medical Center, Phoenix, Arizona, USA). The specimens were provided by the *LifeLegacy Foundation* (Tucson, Arizona, USA), processed within eight hours after death, and cryopreserved at − 80 °C in liquid nitrogen. None of the donors had any known intracranial conditions. All dissections were limited to approach-based exposure of the brainstem surface; no intrinsic parenchymal dissections of the brainstem were performed. The objective was to preserve anatomical integrity while identifying reproducible surface landmarks relevant to SEZs and quantitative exposure analysis. Anterior medullary SEZs were not included in this analysis, as they are not accessed through the far-lateral corridor and fall outside the geometric exposure evaluated in this study.

Upon arrival at the laboratory, the heads were thawed, and the carotid and vertebral arteries, as well as the internal jugular veins, were identified at the cervical sectioned surface and cannulated with silicone tubing of graded diameters, secured with cotton ties (Ethicon Inc., Somerville, NJ, USA). The vessels were flushed under pressure with a clot-removing solution and filled under pressure with red-pigmented silicone (Dow Corning Corp., MI, USA; Crayola Inc., PA, USA) mixed with Catalyst One. The internal jugular veins were filled with blue silicone to highlight the venous system.

Specimens were then immersed at room temperature in sealed containers containing a custom fixative made of 7.5% formalin, methanol, and 80% glycerol (Sigma-Aldrich, MO, USA). This preparation preserved the near-physiological consistency of the skin, muscle, meninges, and brain tissue, enabling realistic microsurgical manipulation.

### Surgical simulation

**Fig. 1:**
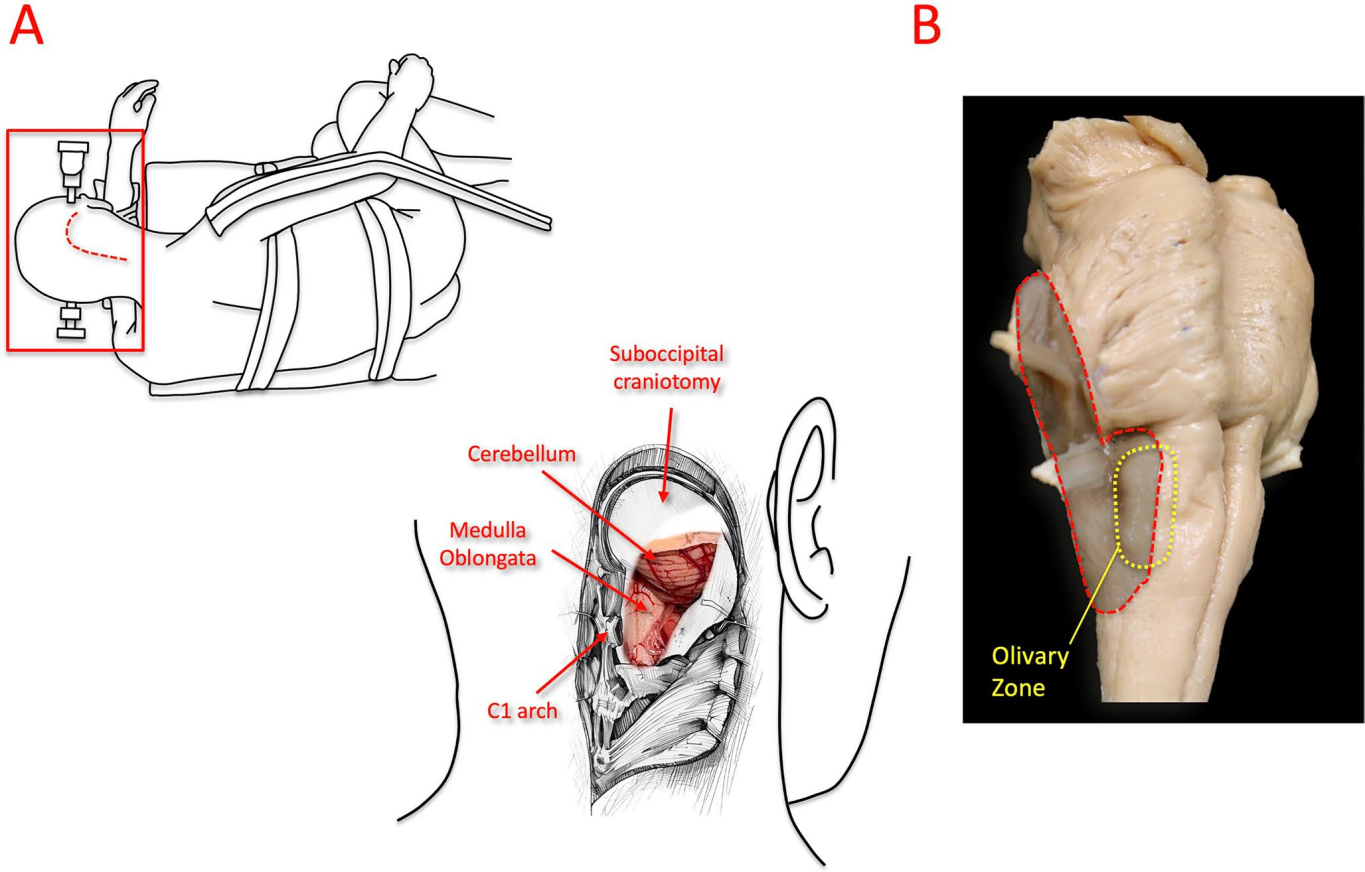
Positioning and surgical exposure for the far-lateral suboccipital approach. (On Left) Simulated park-bench positioning for the far-lateral suboccipital approach, illustrating the patient’s lateral decubitus position with head fixation and the J-shaped skin incision (red dashed line). Only the head was dissected in this study; however, the operative setup was replicated to simulate clinical positioning, as shown within the red frame. A lateral suboccipital craniotomy with partial removal of the posterior arch of C1 exposes the cerebellum, medulla oblongata, and ventrolateral part of the lower brainstem through the far-lateral corridor. (Upper Right) The olivary zone is indicated as the primary anatomical landmark of this exposure, and the red dashed area marks the region of medullary exposure achieved via the far-lateral approach

**Fig. 2:**
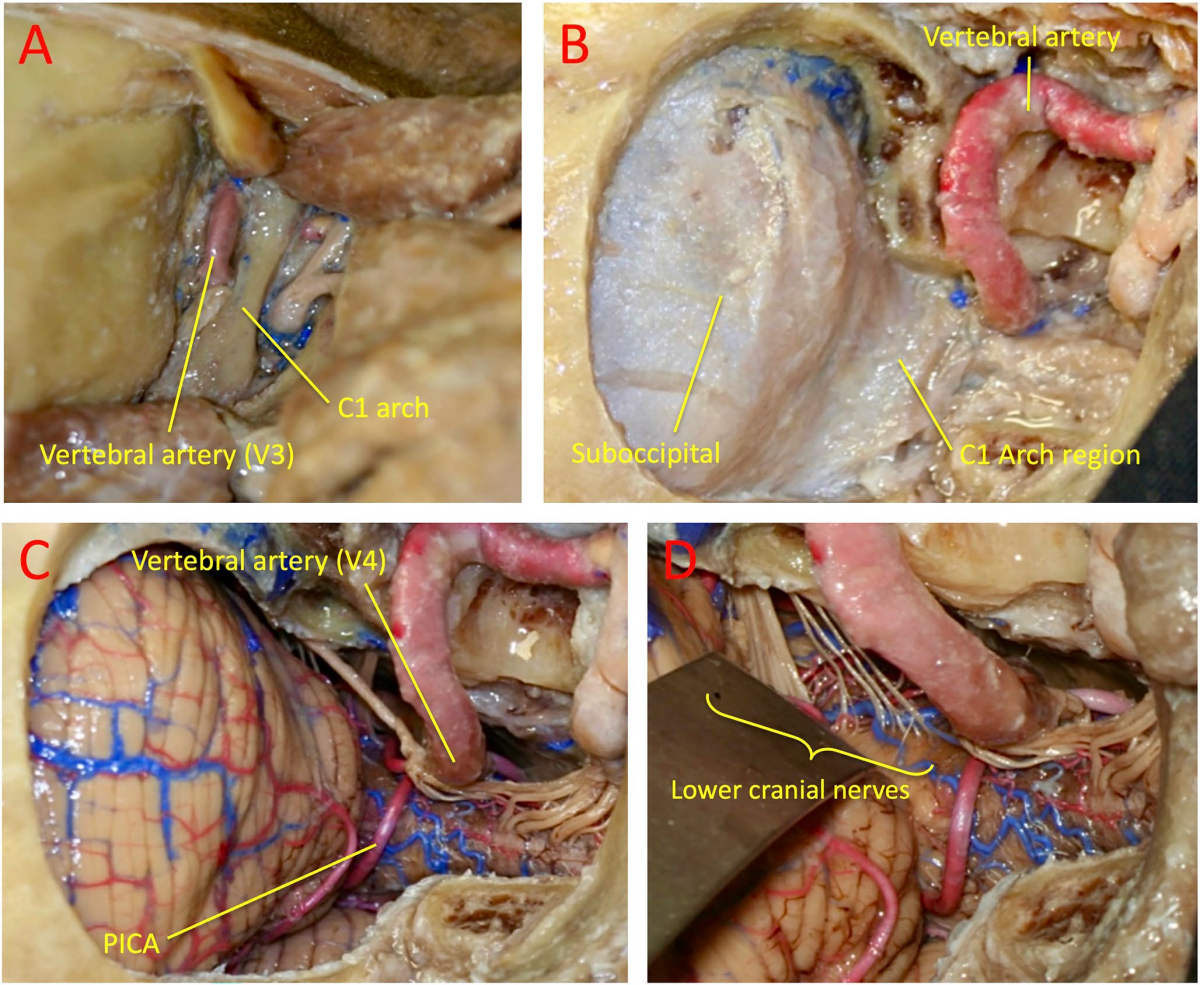
Stepwise dissection and exposure in the far-lateral suboccipital approach. This approach enables assessment of the horizontal and vertical working angles to the olivary region of the medulla. After positioning in the park-bench (three-quarter prone) position, a hockey-stick, L-shaped, or S-shaped incision is performed, followed by careful muscle dissection to expose the suboccipital muscular triangle formed by the superior and inferior oblique and semispinalis capitis muscles. **A**: The suboccipital triangle preserves the V3 segment of the vertebral artery, which is exposed over the posterior arch of C1. **B**: Far-lateral suboccipital craniotomy exposes the posterior margin of the sigmoid sinus and involves partial drilling of the posteromedial third of the occipital condyle. **C**: Dural opening reveals the cerebellum, lower cranial nerves, V4 segment of the vertebral artery, and the posterior inferior cerebellar artery (PICA). **D**: Visualization of the cerebellomedullary fissure and the emergence of the lower cranial nerves (IX, X, and XI)

Each specimen was mounted on a Mayfield head-holder in the operative position according to the planned approach and placed on a surgical table that simulates a standard operating environment. The far-lateral approach was performed on all specimens following surgical steps as in vivo (Figs. 1 and 2). Craniotomies and osteotomies were carried out using an Anspach XMax high-speed drill with various burrs (DePuy Synthes, Palm Beach Gardens, FL, USA). Microsurgical dissections were carried out under an operating microscope—Zeiss OPMI Pentero (up to 40× magnification, Carl Zeiss Meditec, Heidenheim, Germany). Each step was photographed with a Canon Rebel XS 10.1 MP digital SLR camera (Canon, Japan). Selected images were used for publication. The step-by-step progression of the far-lateral approach, from muscular dissection to dural opening and final surface exposure of the medulla and lower cranial nerves, is represented in Fig. 2, which illustrates the sequential anatomical stages used for landmark identification for the measurements. In all specimens, the far-lateral approach was standardized to include partial drilling of the posteromedial one-third of the occipital condyle, a widely accepted modification that optimizes operative freedom while preserving craniocervical stability; no paracondylar, supracondylar, or extreme lateral variants were performed.

## Quantitative measurements

**Fig. 3:**
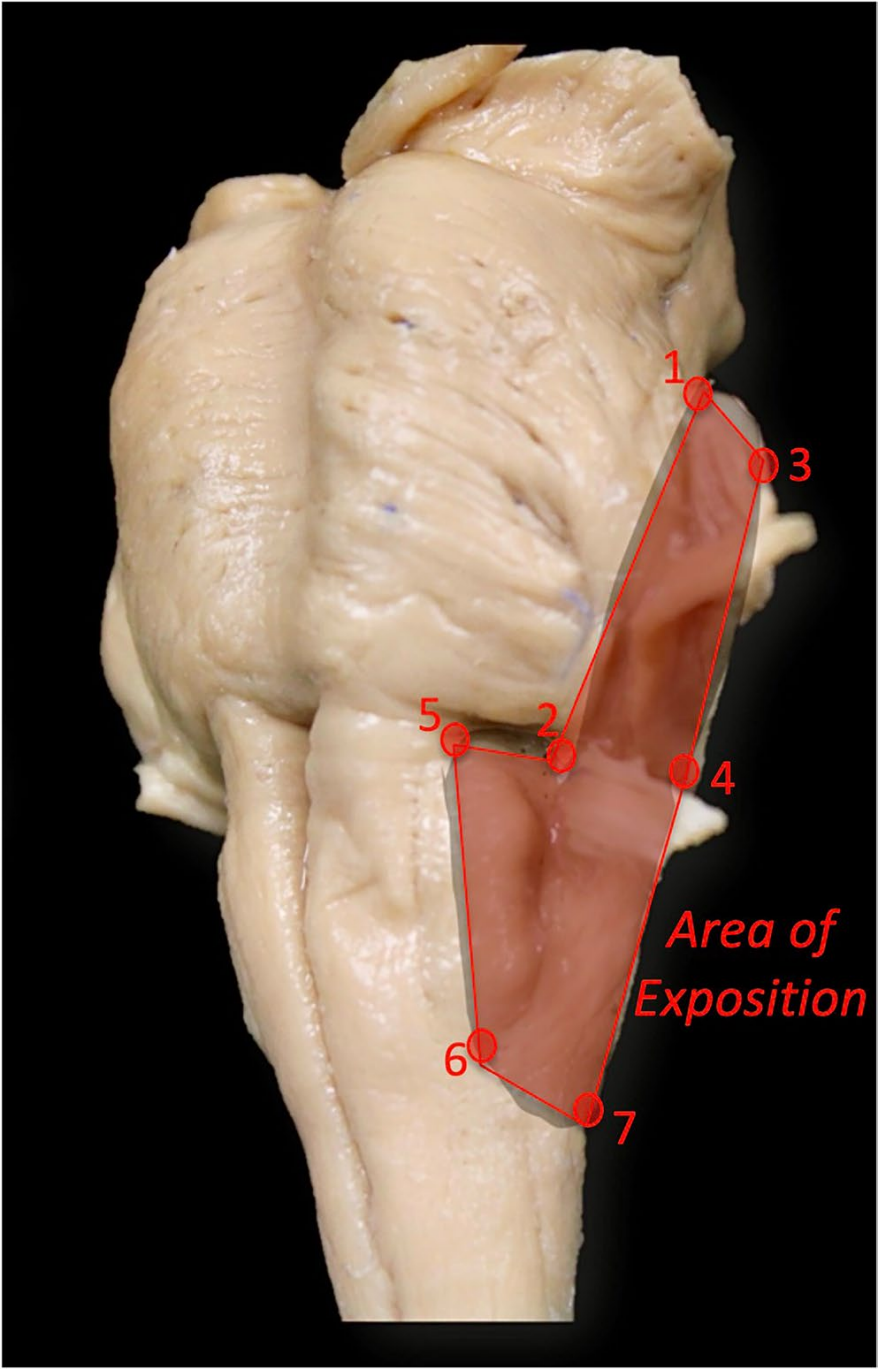
Definition of the exposure area of the lower brainstem, including the pons and ventrolateral medulla*.* The following landmarks delimit the outlined region: (**1**) the most posterior point behind the CN VII–VIII complex; (**2**) the most posterior point on the pons; (**3**) the most anterior and cranial point on the pons; (**4**) the most anterior and caudal point on the pons; (**5**) the most cranial point on the ipsilateral medulla; (**6**) the most caudal point on the ipsilateral medulla; and (**7**) the ipsilateral mirror point to landmark 6. Landmark coordinates were obtained by direct placement of the neuronavigation probe on the exposed brainstem surface, without image-based registration, and were used to reconstruct the exposure polygon

Once the far-lateral approach was performed, three-dimensional (3D) coordinates were obtained using a neuronavigation system (StealthStation Treon Plus; Medtronic Navigation, Louisville, CO, USA) to identify key anatomical landmarks on the brainstem surface. The exposure area was defined by seven reproducible points: (1) the most posterior point behind the CN VII–VIII complex; (2) the most posterior point on the pons; (3) the most anterior and cranial point on the pons; (4) the most anterior and caudal point on the pons; (5) the most cranial point on the ipsilateral medulla; (6) the most caudal point on the ipsilateral medulla; and (7) the ipsilateral mirror of point 6 (Fig. 3).

**Fig. 4:**
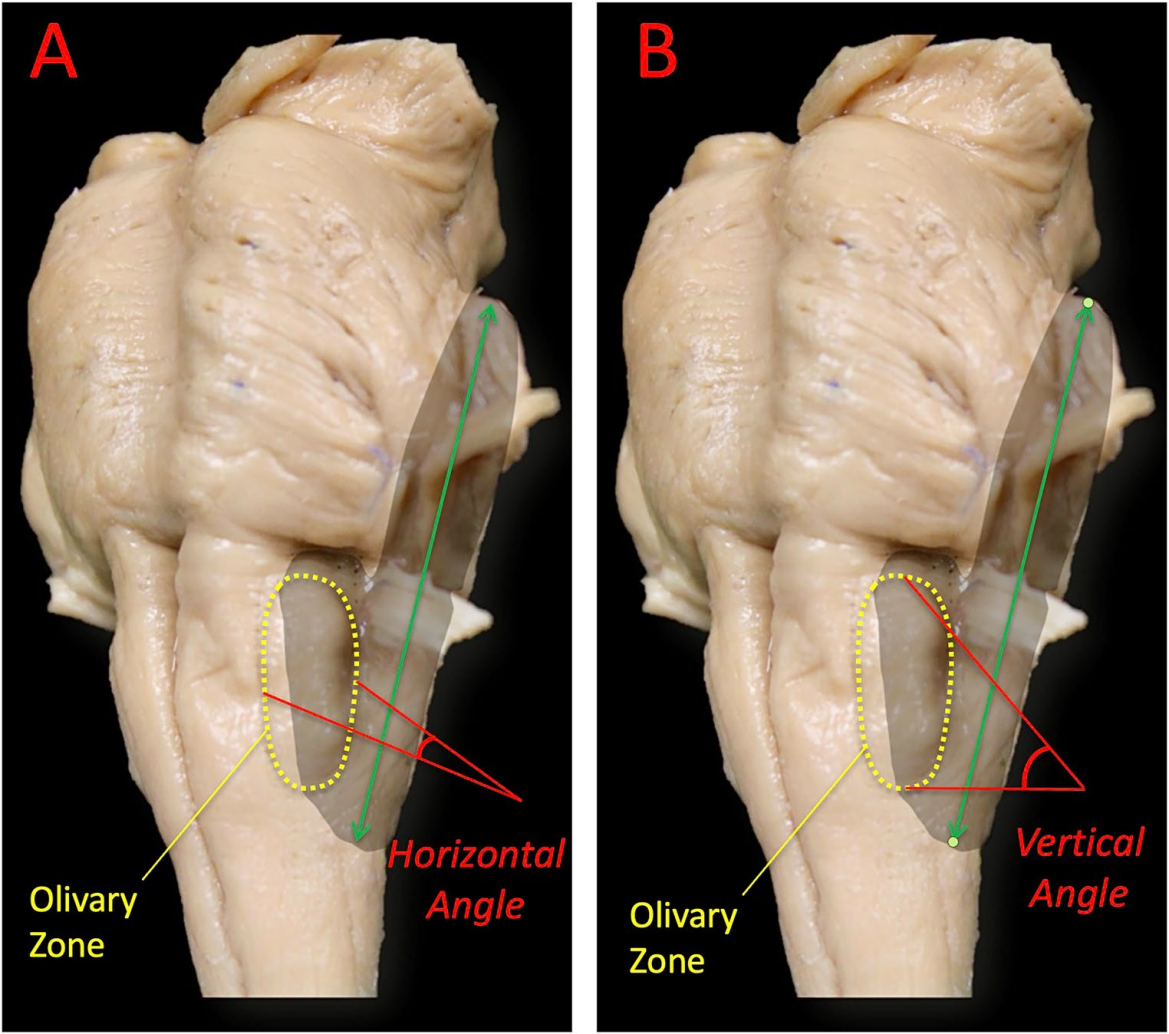
Microsurgical working angles to the safe entry zone on the medullary olive*.* **A**: Horizontal working angle defined by the anterior-most and posterior-most limiting points on the olive, representing the optimal mediolateral trajectory for safe exposure. **B**: Vertical working angle projected along a line from the pontomedullary junction to the most caudal point of the olive, delineating the superior–inferior trajectory for controlled access to the ventrolateral medulla. The green arrow represents the vertical linear extent of exposure achieved through the far-lateral approach

Using these coordinates, the surgical working area was calculated with customized Microsoft Excel (Microsoft Corp., Redmond, WA) formulas that created 3D polygons projected onto a two-dimensional plane for area measurement. The horizontal and vertical working angles toward the olivary (ventrolateral medullary) zone were determined using Spherical Area Software (BitWise Ideas Inc., Fredericton, Canada), which allowed for the quantification of trajectories and linear extensions across all three spatial planes. Additionally, the vertical linear extent of exposure—defined as the maximum measurable distance along the vertical working axis from the pontomedullary junction to the caudal limit of the olive—was calculated to represent the superior-inferior reach of the far-lateral approach (Fig. 4).

Mean values and standard deviations for the exposure areas and angular measurements were recorded in Excel for subsequent quantitative and comparative analysis.

In this study, neuronavigation was used exclusively as a three-dimensional spatial digitization tool. No MRI or CT datasets were acquired or registered. After completion of the far-lateral exposure, the navigation probe was placed directly on predefined surface landmarks of the exposed brainstem, and the corresponding 3D coordinates were recorded to enable geometric reconstruction of the exposure area, working angles, and linear extent.

The university’s Ethics Committee approved the microsurgical cadaveric dissections. This study was based on cadaveric dissections and previously published studies and did not require informed consent from patients, as no new human or critical data were collected.

## Results

### Medullary safe entry zones

**Fig. 5:**
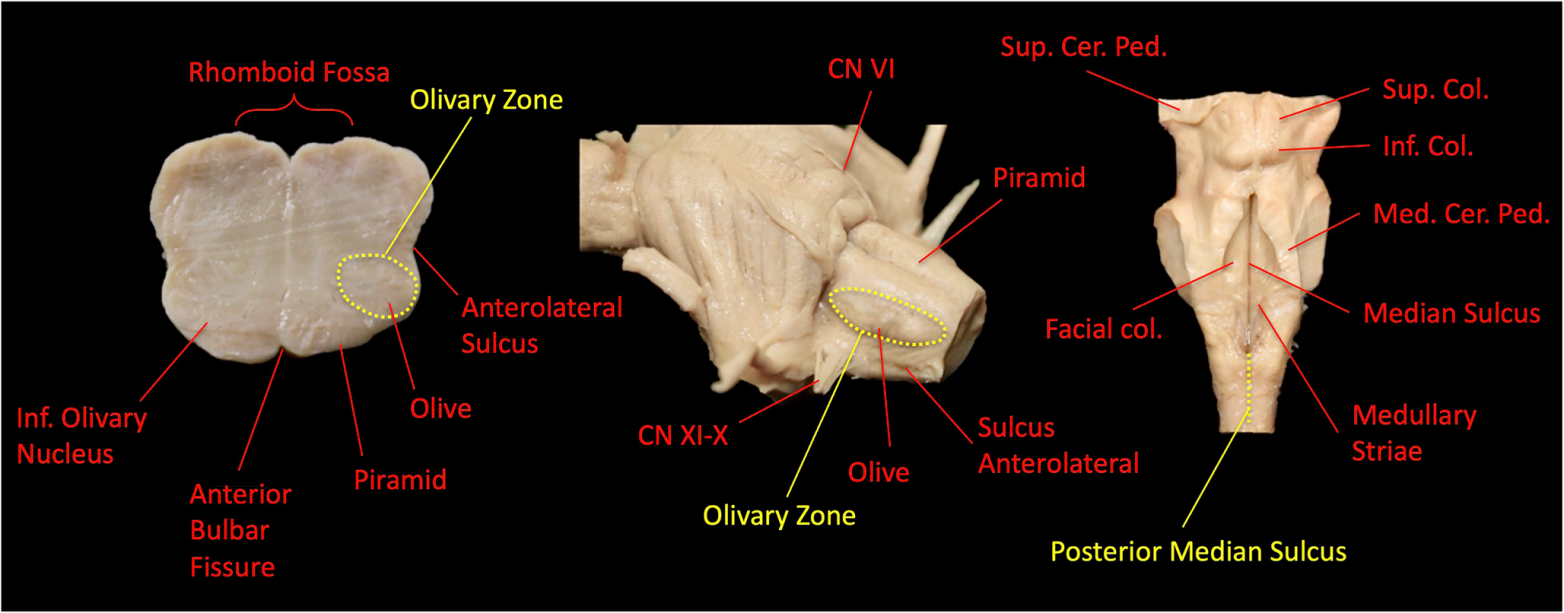
Anatomical landmarks of the medulla oblongata and identification of safe entry zones. The posterior median sulcus and the olivary zone serve as safe entry pathways to the dorsal and ventrolateral medulla, respectively. The figure illustrates the inferior olivary nucleus, the olive, the anterolateral sulcus, and the bulbopontine sulcus, delineating the relationship between the rhomboid fossa, the pyramid, and surrounding structures relevant to the selection of a surgical approach

Based on the literature and our dissections, the principal SEZs of the medulla oblongata relevant to our approaches were the olivary region (ventrolateral medulla), which is anatomically delimited by the pre-olivary sulcus anteriorly, the post-olivary sulcus posteriorly, and the posterior median sulcus. This ‘delimitation’ describes surface anatomy for navigation and exposure mapping; the operative entry point and trajectory remain lesion-dependent (Fig. 5). These corridors were consistently identifiable and provided reproducible surface landmarks for trajectory planning to the ventrolateral and dorsal medulla, respectively [2, 9, 10].

### Surgical approach to the medulla oblongata

In this study, the far-lateral approach was anatomically evaluated and quantitatively analyzed as the primary corridor to the ventrolateral medulla and the olivary region. The midline route provided direct access to the posterior median sulcus and, depending on tonsillar and vermian relationships, to the supra- and infracollicular zones of the floor of the fourth ventricle. The far-lateral route provided optimized access to the ventrolateral medulla and the olivary zone, facilitated by partial removal of the C1 arch and limited condylar drilling when necessary. Table 1 summarizes the mapping between approaches and SEZs [2, 7–10].

**Table 1:** Principal safe entry zones of the medulla oblongata and their commonly associated surgical approaches, based on anatomical exposure and previously published literature

Surgical approach	Safe entry zone	Abbreviations/Landmarks
Far-Lateral	Olivary Zone	ZO = Olivary Zone
Midline Suboccipital	Posterior Median Sulcus, Supracollicular Zone, Infracollicular Zone	MP = posterior median sulcus of the medulla; ZSC = supracolicular zone; ZIC = infracolicular zone

Abbreviations: *MP* posterior median sulcus of the medulla, *ZSC* supracolicular zone, *ZIC* infracolicular zone, *ZO* olivary zone

## Quantitative exposure of medulla oblongata (far-lateral approach)

Neuronavigation-based measurements outlined a polygonal exposure area on the lower brainstem using seven reproducible landmarks that span the pons and ipsilateral medulla **(**Fig. 3**)**. For the far-lateral approach, the average exposed area was 856.8 ± 139.7 mm². The horizontal and vertical working angles toward the olivary (ventrolateral medullary) zone averaged 40.8 ± 10.2° and 54.8 ± 6.8°, respectively, with a mean vertical linear extent of 60.0 ± 6.6 mm along the working axis (Fig. 4; Table 2). These data quantify the maneuverability of instruments and the microscope’s line of sight to the olivary target achievable through the far-lateral corridor.

**Table 2:** Surgical exposure area provided by the far-lateral approach to the ventrolateral region of the medulla oblongata. Values represent mean ± standard deviation (SD) of the quantitative parameters measured using neuronavigation after cadaveric dissection

Area (mm^2^)	Mean ± SD
	856.8 ± 139.7
Horizontal angle (^o^)	40.8 ± 10.2
Vertical angle (^o^)	54.8 ± 6.8
Vertical linear extent (mm)	60.0 ± 6.6

## Discussion

Our qualitative and quantitative analysis provides operative planning metrics for exposing the lower brainstem—specifically, the ventrolateral medulla (olivary region)—using the midline suboccipital and far-lateral approaches. These results fit within, and expand upon, the anatomical framework of safe-entry zones (SEZs) that have guided brainstem surgery for over a decade [11]. Anatomical studies have identified reproducible pathways on the anterolateral and dorsal brainstem where critical nuclei, long tracts, and perforators are relatively sparse; among these, the olivary zone and posterior median sulcus are most relevant to medullary lesions [2, 9, 12]. By mapping a polygon of surface landmarks and calculating horizontal and vertical working angles, we turn descriptive anatomy into quantitative exposure, aiding in the practical selection of approach and trajectory to reach the olivary SEZ while minimizing parenchymal transgression [13].

Although this study is based on surface exposure and quantitative geometry, the ventrolateral medullary corridor analyzed here is anatomically related, at depth, to the inferior olivary nucleus, the adjacent reticular formation, and nearby long tracts, including the corticospinal, spinothalamic, and spinocerebellar pathways. This region is closely associated with perforators arising from the vertebral artery and posterior inferior cerebellar artery, which are critical during surgical entry. The present work focuses on surface anatomy and exposure geometry; detailed surface-to-depth correlations of nuclei, tracts, and vascular microanatomy have been extensively described in prior anatomical studies and are beyond the scope of this quantitative exposure-based analysis.

Approach selection must balance lesion topography with the shortest, safest route—the core principle of the “two-point method”—without losing the benefits of entering through an SEZ [8]. In our series, the midline suboccipital approach favored posterior midline corridors (posterior median sulcus, supra-/infracollicular zones), while the far-lateral approach optimized access to the ventrolateral medulla and lower cranial nerve complexes. This reflects current practice algorithms that combine the two-point strategy with SEZs and image guidance to avoid tangential trajectories and reduce retraction-related morbidity [8]. Our measurements also align with posterolateral skull-base data, indicating that far-lateral extensions extend caudal reach and improve access to the lower clivus and lower brainstem—trade-offs that must be weighed against additional drilling and vertebral artery handling [14, 15]. Accordingly, SEZs should be interpreted as relatively low-risk corridors rather than fixed safe sulci, and their practical use depends on lesion-driven distortion of surface landmarks and tract displacement.

From a corridor-based and ergonomic standpoint, alternative approaches to the medulla oblongata have inherent limitations in addressing ventrolateral lesions. The midline suboccipital approach, while optimal for dorsal and posterior midline entry zones, imposes an oblique, elongated trajectory to the ventrolateral medulla, with restricted lateral working angles and less favorable microscope ergonomics. Similarly, the retrosigmoid approach offers limited inferior and anterior reach to the lower ventrolateral medulla because of constraints from the petrous bone, sigmoid sinus, and cerebellar retraction. In contrast, the far-lateral approach aligns the surgical corridor more directly with the ventrolateral medulla, improving horizontal and vertical working angles and cranio-caudal reach, as demonstrated by the quantitative exposure parameters in this study.

Clinical series confirm that when these corridors are used carefully—especially for cavernous malformations—the surgical morbidity can be comparable to the natural history. Large, single-center reports have shown durable resections with acceptable long-term outcomes, especially when lesions are exophytic, pial-presenting, or near an SEZ; careful case selection remains crucial for midline pontine and deeply located lesions [1]. Additionally, institutional studies emphasize that anterolateral routes to the pons and ventrolateral medulla can reduce the incidence of permanent new deficits compared to dorsal trans-fourth ventricle entries in selected cases [16]. Our quantitative analysis of working angles and the vertical linear extent of exposure (along the pontomedullary-to-olive axis) offers objective metrics that can help standardize the planning and execution of far-lateral exposures, aiming to reproduce these positive clinical results.

Intraoperative neurophysiology further supports the safe crossing of SEZs. Mapping (facial colliculus; hypoglossal and glossopharyngeal triangles) helps identify entry points when the fourth ventricle floor is distorted, and continuous monitoring of corticospinal and corticobulbar pathways detects evolving risks that anatomical landmarks cannot. Although techniques for corticobulbar motor-evoked potentials are still developing and not yet fully standardized, their integration with free-running EMG and brainstem mapping has clearly reduced postoperative cranial nerve morbidity in complex posterior-fossa surgery [17]. These methods should be routine assistants whenever pathways approach the lower cranial nerve nuclei or cross the olive.

Pediatric experience provides an additional perspective: despite differences in pathology mix and biomechanics, large pediatric series show that disciplined use of SEZs, careful skull-base exposure (often far-lateral), and thorough monitoring enable meaningful resections with low mortality and acceptable morbidity. This supports the general applicability of SEZ-guided strategies across age groups and affirms the external validity of our quantitative medullary corridors [5].

Limitations include the cadaveric nature of our measurements (no edema, tumor mass effect, or vascular engorgement) and the use of a single laboratory methodology. Nevertheless, our protocol—physiologic tissue preparation, navigation-based coordinates, and explicit landmark definition—closely mirrors prior anatomic standards and provides reproducible metrics [2, 9]. Future research should combine these measurements with patient-specific imaging (tractography, lesion growth vectors) and prospective outcome registries to validate how exposure area, angle, and vertical linear extent of exposure predict operative ergonomics, extent of resection, and neurological outcomes.

## Conclusion

This anatomical and quantitative analysis defines the far-lateral approach as a geometrically efficient and reproducible corridor to the ventrolateral medulla and olivary safe entry zone. By integrating neuronavigation-based measurements with microsurgical dissection, we objectively characterized the exposure area, working angles, and vertical linear extent achievable through this approach, thereby transforming descriptive medullary anatomy into measurable operative parameters. The far-lateral route offers favorable horizontal and vertical trajectories and significant cranio-caudal reach along the pontomedullary axis, supporting its role as the preferred approach for intrinsic lesions centered within or extending toward the olivary region. These data refine surgical planning by outlining the spatial limits and maneuverability inherent to the far-lateral corridor and reinforce the anatomical rationale for using it when direct anterolateral access is required to minimize transgression of eloquent nuclei and long tracts. When combined with modern image guidance and intraoperative neurophysiological monitoring, this quantitative framework may contribute to more precise, standardized, and safer microsurgical access to lesions of the medulla oblongata.

## Data Availability

No datasets were generated or analysed during the current study.
